# The Lens–patent and scholarly search analysis

**DOI:** 10.29173/jchla29842

**Published:** 2025-12-01

**Authors:** Debbie Chaves

**Affiliations:** Head, Copyright and Course Resources Wilfrid Laurier University Waterloo, ON, Canada

**Product:** The Lens–patent and scholarly search analysis

**URL:** https://www.lens.org

**Owned by:** Cambia (cambia.org) from Australia as a non-profit.

## Product description/purpose

The Lens is a free open platform offering links to the full-text of results. Founded in 2000 as a free open patent search engine, The Lens has grown into a database offering patent and biomedical patent biological sequence information, policy, data, and research articles [1,2].

## Intended audience/users

The open accessible nature of this platform allows for a diverse set of users, such as users searching for patent information and academic literature. The large corpus, ease of use, and the ability to export mean that this database can be used for quick health questions and knowledge synthesis [3].

## Special features

### Sources

**Fig. 1:**
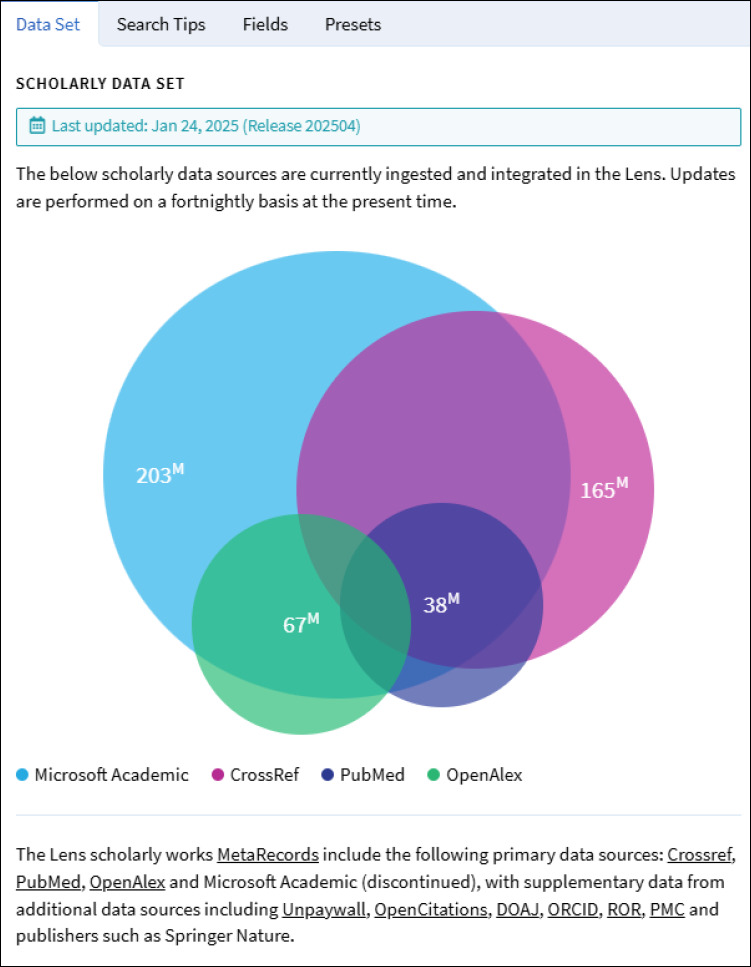
Screenshot of scholarly works contained in Lens.org. See the last updated date included. The content is updated regularly.

The Lens uses data from multiple sources including Microsoft Academic (now defunct), CrossRef, OpenAlex, and PubMed. This creates a large multi-disciplinary database that contains millions of sources (Figure 1) [4].

### Citation analysis

Yes, it includes citation analysis of both scholarly works and patents. Individual citation metrics can be viewed in The Lens “Profiles” which uses ORCID as a persistent identifier. The quick ability to provide citation graphs and visualizations is helpful in connecting papers together and means that information does not have to be exported from the search database into VosViewer or another platform for network analysis. The Lens rated highly for forward citation searching, which can be useful in systematic reviews [5].

### Advanced search options

**Fig. 2:**
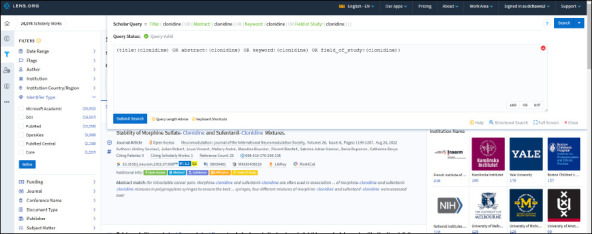
Advanced query status offers comprehensive Boolean searching

Boolean searching is available and best visible in the “Query Text Editor” offering to create complex search strategies. See Figure 2 for a screenshot of a complex search for “clonidine” which produced a total of 24 074 results for which 65% had a PubMed ID. It may be that the metadata from PubMed was faulty thus a more detailed analysis of duplicates is necessary, but the general observation is that the database is larger than searching just PubMed. The Lens offers a “Search History” feature that is useful in addition to the “Saved Queries” feature [6].

### Use of artificial intelligence

The Lens uses generative AI (GenAI) for searching. It employs text mining and data analysis to enhance the search results dashboard. In addition, the “Subject Matter” filter combines “Subject”, MeSH and “Field of Study” through GenAI. The energy consumption that most GenAIs require should be a consideration when using a database that incorporates GenAI [7].

### Filtering

Filters are available to narrow down the search results (Figure 2). Significantly, there is no filter for language as OpenAlex does not have metadata for language. Dashboards are created by the GenAI in The Lens to display the search results using the “Analytics” tab and can be shared externally allowing easy connections.

## Compatibility

The Lens is compatible with a wide array of browsers.

## Usability/user interface

**Fig. 3:**
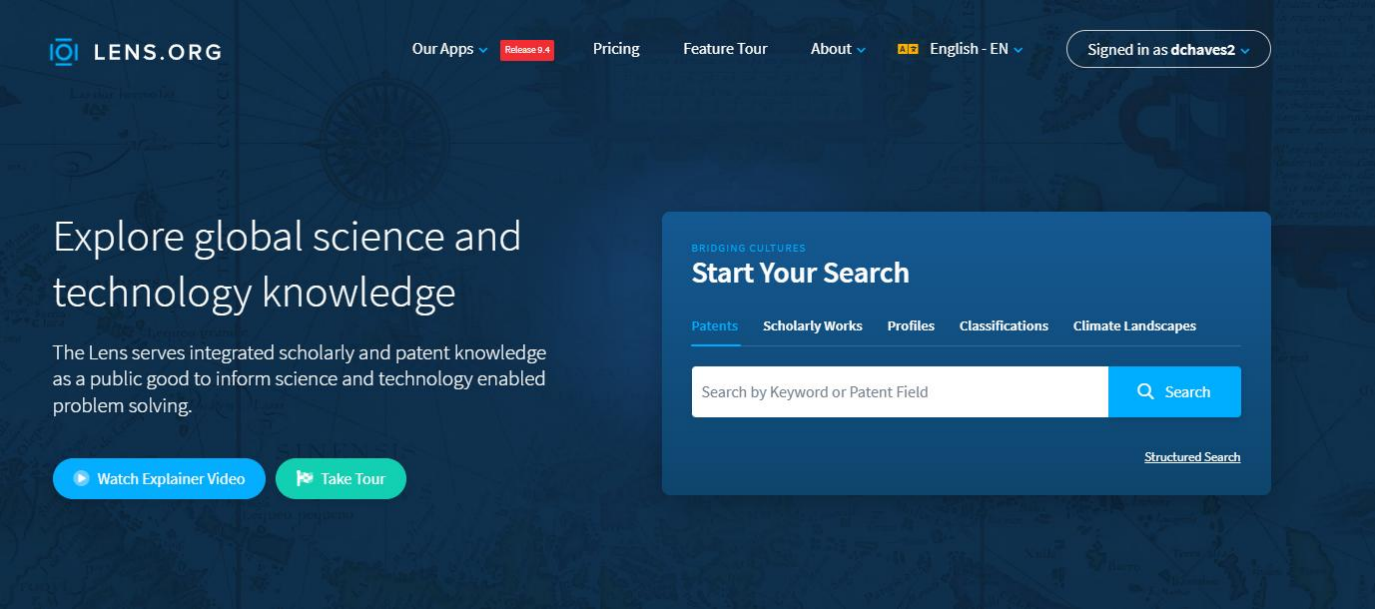
Landing page for The Lens (https://www.lens.org)

You can log in using a variety of methods; however, if you log in using an ORCID iD, you can integrate your ORCID information into The Lens and create a profile. Accessing the full search page is a little confusing (Figure 3). The landing page defaults to a patent search box. Click on the “Scholarly Works” tab and you can leave the search box blank. Then click “Search” or click the “Structured Search” button beneath the search box to fully access the database. Finding the link to the full-text of results can also be a bit tricky. Scroll down the page and look to the far right. The full-text can be found by clicking within the “Open Access” box or the “Sources” box.

## Strengths and weaknesses

The Lens is a large multidisciplinary database with ease of use for both quick searches and more in-depth search strategies. Overall, looking for quick searches, I tend to use the newer GenAI databases like Elicit (https://elicit.com/welcome) or Consensus (https://consensus.app/) as they provide quick summaries. The Lens would be useful as a large multidisciplinary database to search for systematic reviews. Search strings would need to be translated from other databases. In general, I use this database for bibliometric analysis of my institution and to provide easy links to the data visualizations available in the dashboard. Others use the database for promotion of staff publications [8]. The database itself has many strengths, but it does not seem to be very popular, and many users are not familiar with its availability.

## Comparison with similar products

Due to the citation analysis ability, The Lens can be compared to Elsevier’s Scopus, Clarivate’s Web of Science, Digital Science’s Dimensions (https://www.dimensions.ai/), OpenAlex (https://openalex.org/), and Google Scholar [9]. The Lens is the largest database of journal article records with comprehensive metadata as reported by The Lens itself [10], although this is debated by Delgado-Quirós & Ortega [11]. The Lens, OpenAlex, Scopus, and Dimensions allow APIs where Web of Science does not. The Lens has great user design and functionality. While The Lens includes data from PubMed, it does not replicate all the specific domain filters or curated indexing depth of PubMed. Like OpenAlex, The Lens has some difficulty with affiliation information compared to the highly curated Scopus and Web of Science commercial platforms [12].

## Currency/period covered

From 1800 onwards. Latest Release: Version 9.6, May 22, 2025, while the previous release was March 3, 2025 [13].

## Privacy

You will have to provide information such as name and email address to activate the account. The Lens uses cookies and collects information regarding the browser, language, and location, but this information will not be shared without your permission. They allow a variety of users including anonymous, individual, and institutional [14].

## Cost/value

The Lens is free and open with abstracts and links to full-text. Cambia pledges that The Lens will be free with no premium options as quoted on their webpage: “With us, everyone can access and use the website without an account at no cost. We think the ability for anyone to create new value should not be constrained by access to critical knowledge” [3]. Additional monies are needed for individuals and institutions that are profit-based. The Lens offers value in being free and open with extensive data sources and powerful features and excels in analytics and visualization tools for a free platform.

## Contact information

- Website: https://www.lens.org
- For support: dpo@lens.org or support@lens.org or directly:
  - The Lens Data Protection Officer, Lens@QUT 133 Wickerslack Lane Karabar, NSW 2620, Australia
- Address: The Lens Cambia GPO Box 3200 Canberra, 2601, Australia
